# Evaluation of miniscrew angulation in the posterior maxilla using cone-beam computed tomographic image

**DOI:** 10.1590/2177-6709.23.1.046-053.oar

**Published:** 2018

**Authors:** Henrique M. Villela, Mario Vedovello, Heloísa C. Valdrighi, Milton Santamaria-Jr, Carolina Carmo de Menezes, Silvia A. S. Vedovello

**Affiliations:** 1 Uniararas, Fundação Hermínio Ometto, Programa de Pós-graduação em Ortodontia (Araras/SP, Brazil).

**Keywords:** Bone screw, Implants, Tomography, Orthodontics

## Abstract

**Objective:**

This study aimed at evaluating whether changes in the insertion angle is a determining factor in the positioning of the miniscrews body in a region with larger interradicular space in the posterior maxilla.

**Methods:**

Analysis of 60 posterior maxillary quadrants were made using images obtained by means of cone-beam computed tomographic image (CBCT), with 0.076-mm voxel, which presented a real miniscrew inserted in the mesial region of the maxillary first molars, serving as reference point for the placement of the virtual miniscrews. Measurements of the distances between roots were made in three points on the body of the virtual miniscrews (A, B and C), at four different angulations, 70^o^, 60^o^, 50^o^ and 40^o^ (T_1_ to T_4_), in relation to the long axis of the second premolar. This evaluation was made in four groups, selected in accordance with the disposition of the roots of the second premolars and first molars: Group 1 (all types of roots), Group 2 (convergent roots), Group 3 (divergent roots) and Group 4 (parallel roots).

**Results:**

There were no statistically significant differences in the measurements of points A, B and C, at the different angles (70^o^, 60^o^, 50^o^ and 40^o^) and in the different groups (p > 0.05).

**Conclusions:**

Changes in the insertion angle is not a determinant factor in the positioning of miniscrews body in regions with larger interradicular space in posterior maxilla.

## RESUMO

**Objetivo:**

o presente estudo objetivou avaliar se alterações no ângulo de inserção do miniparafuso são um fator decisivo para a adaptação do corpo do parafuso em áreas com espaço inter-radicular aumentado, na região posterior da maxila.

**Métodos:**

foram realizadas análises em 60 quadrantes maxilares posteriores, a partir de imagens obtidas por tomografia computadorizada de feixe cônico (TCFC), com voxel de 0,076 mm, as quais apresentavam um miniparafuso real inserido na região mesial dos primeiros molares superiores, utilizado como ponto de referência para a inserção de miniparafusos virtuais. As distâncias inter-radiculares foram mensuradas utilizando-se três pontos marcados no corpo dos miniparafusos virtuais (A, B e C), inseridos com quatro angulações diferentes (70°, 60°, 50° e 40°) (T_1_ a T_4_) em relação ao longo eixo do segundo pré-molar. A avaliação foi realizada em quatro grupos, selecionados de acordo com a disposição das raízes dos segundos pré-molares e primeiros molares: Grupo 1 - contendo todos os tipos de raízes; Grupo 2 - raízes convergentes; Grupo 3 - raízes divergentes; e Grupo 4 - raízes paralelas.

**Resultados:**

não houve diferença estatisticamente significativa entre as medidas dos pontos A, B e C nas diferentes angulações (70°, 60°, 50° e 40°) ou entre os grupos (p > 0,05).

**Conclusões:**

alterações no ângulo de inserção dos miniparafusos não são um fator decisivo para a adaptação do corpo do parafuso, em áreas com espaço inter-radicular aumentado entre primeiro molar e segundo pré-molar superiores.

## INTRODUCTION

Miniscrews have brought a new perspective to orthodontic treatment due to their low cost, effectiveness and easy clinical management.^1^ ^,^ ^2^ ^,^ ^3^ Although the procedure for miniscrew insertion is simple, some care must be taken with the purpose of minimizing the risks of the miniscrew body contacting the roots of teeth, such as: evaluating the bone availability in the interradicular space; use of simplified surgical guides, and the use of a safe surgical protocol.^1^ The possibilities of causing damage to periodontal structure and the roots must not be underestimated. Among the problems can be included: displacement of bone into the periodontal ligament space; damage to cement; damage to dentin and pulp damage.^4^ ^-^ ^7^

One of the factors that may vary during miniscrew insertion into the maxilla is angulation, which may be more perpendicular or more angulated in relation to the vestibular bone cortical surface, or in relation to the long axis of the teeth. Some authors have recommended a more perpendicular insertion into the maxilla, since this factor would diminish the risk of the screw body contacting the roots, and generates a line of action of force closer to the center of resistance.^8^ Other authors have recommended miniscrew insertion into the maxilla with an angulation of 30^o^ to 40^o^ in relation to the long axis of the tooth, with the purpose of minimizing the risks of the screw contacting the roots.^9^ ^-^ ^11^

Primary stability may be increased when the miniscrew is inserted at angles of 60^o^ to 70^o^ in relation to the bone surface, in regions with thicker cortical bone, but for this purpose a higher torque is demanded for its insertion. However, this increased angulation may cause a higher failure rate due to excessive pressure on the bone.^12^ The bone density in the posterior region of the maxilla is lower than it is in the mandible, and this area also presents a thin vestibular cortical.^13^ ^-^ ^15^ Studies in the mandibles of human cadavers and using finite elements method have shown that the insertion of screws at 90^o^ in relation to the bone surface offered greater resistance and less stress on cortical bone than that of screws inserted at 60^o^ and 30^o^.^16^ Stability and resistance to failure do not depend on the orientation of miniscrew implantation in relation to the bone surface, however, miniscrews inserted at 90^o^ presented greater stability in shear tests, in comparison with those inserted at 45^o^. This higher degree of stability occurred due to the line of action of force being positioned closer to the long axis of screws perpendicular to the bone surface.^17^ Screw insertion in a more apical and angled position not only increases the risk of contact with the maxillary sinus, but also increases the risk of sliding during its insertion.^18^ The miniscrew may be placed at an angle between 55^o^ and 70^o^ in relation to the occlusal plane, in the infrazygomatic crest region, above the maxillary first molar, with the purpose of preventing its contact with the root. However, in order for this strategy to be efficient, the miniscrew must be inserted at a distance of 14 to 16mm from the occlusal plane.^19^ One of the greater risk factors of this anchorage system is inflammation of the peri-screw soft tissues, which occurs when screws are inserted into the alveolar mucosa. To prevent this from occurring, the screws must preferably be inserted into keratinized mucosa.^20^ ^,^ ^21^

During planning of screw insertion into the molar region, the location of the maxillary sinus must be observed and it perforation prevented, since it could lead to complications such as sinusitis and mucosal retention cysts.^18^ ^,^ ^22^ In-depth knowledge of anatomic relations between the roots and adjacent structures, with the use of tomography, is essential to prevent root injuries; and studies to evaluate which would be the best position of the miniscrew to diminish the possibility of contact with the roots, are of fundamental importance. This is because the proximity of the miniscrew to the root is a risk factor that may lead to the loss of stability and consequent failure of this device as an orthodontic anchorage.^23^ ^,^ ^24^ Studies conducted in human maxillae and mandibles, and studies in tomographies have concluded that the region between the maxillary first molar and second premolar, from the vestibular direction, represent the safest region for performing the insertion of miniscrews at the height of 6 to 8 mm from the cervical line.^9^ ^,^ ^24^

The objective of this study was to evaluate if the change in insertion angle is a determining factor in the positioning of the miniscrews body in a region with larger interradicular space in the posterior maxilla, using real miniscrews inserted in this region with clinical success, as guidance. 

## MATERIAL AND METHODS

This study received approval from a Ethics Committee (FHO/Uniararas, protocol # 2.081.877). The convenience sample comprised images selected from a file containing orthodontics records. These had been captured by means of cone-beam computed tomographic image (CBCT) with 0.076-mm voxel, in a Kodak 9000 3D tomograph (Kodak Dental Systems, Carestream Health, Rochester, NY, USA), which belonged to the file of one of the researchers, according to the following inclusion criteria:

» Brazilian patients of both genders.
» Who had undergone corrective orthodontic treatment.
» With real miniscrews inserted between the maxillary first molars and second premolars at an advanced stage of leveling.
» With real miniscrews inserted by the same clinician professional.
» With CBCT taken by the same tomograph.

The CBCT images of patients who did not fall within the selection criteria were excluded. The final sample consisted of 60 maxillary posterior quadrants of 35 patients, with 26 being female and 09 male, and 30 quadrants from the right and 30 from the left side. 

**Figure 1:**
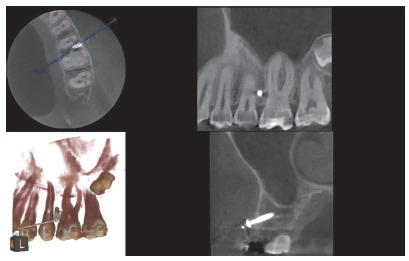
CBCT visualization of three slices and 3D reconstruction.

**Figure 2:**
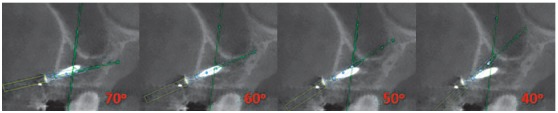
Virtual miniscrews at 70^o^, 60^o^, 50^o^ and 40^o^ of inclination in relation to the long axis of the second premolar, in the transaxial slice.

The CBCT depicted miniscrews previously inserted in the mesial region of the maxillary first molars, which served as reference for positioning the virtual miniscrews (Fig 1). The virtual miniscrews were created, coinciding with the real miniscrews in the vestibular cortical region. This site represents the point of introduction of the miniscrew into the cortical bone and was chosen according to the orthodontic planning. Usually, this site is found in the region of keratinized mucosa, which determines the limit in height for the insertion of a miniscrew. This height may range between 6 to 8mm apical to the line of the orthodontic arch. The virtual miniscrews were created with four different angulations: 70^o^, 60^o^, 50^o^ and 40^o^, in relation to the long axis of the second premolar (Fig 2).

**Figure 3:**
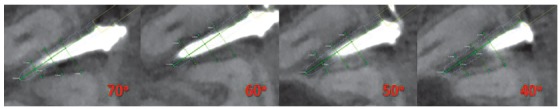
Measurement of the distances between the root of the molar and premolar at points (A), (B) and (C), located at 2.0 mm, 4.0 mm and 6.0 mm, respectively, from the tip of the miniscrew at 70^0^ (T_1_), 60^0^ (T_2_), 50^0^ (T_3_) and 40^0^ (T_4_) of inclination in relation to the long axis of the second premolar, in the transaxial slice.

The insertion and changes in angulation of the virtual miniscrews were performed by means of a specific software program (CS 3D imaging software, Kodak Dental Systems, Carestream Health, Rochester, NY, USA), which is a visualizer of CBCT images in DICOM (Digital Imaging and Communications in Medicine) format. The distance between the roots of the first molars and second premolars was evaluated in three specific points on the body of the virtual miniscrews, on a slice constructed parallel to the long axis of each virtual inclination. These three points were determined by measuring 2mm, 4mm and 6mm from the point of the virtual miniscrews, which presented a body length of 8mm. The measurements were determined on a slice constructed parallel to the long axis of each virtual inclination. The points were denominated as follows: A) 2mm, B) 4mm and C) 6mm from the tip of the miniscrew, respectively. These three distances were measured at the four angulations of the miniscrew, determining: T1 = 70^0^, T2 = 60^0^, T3 = 50^0^ and T4 = 40^0^ (Fig 3). Then, they were duly recorded in accordance with their location (A, B and C) and the angle (70^o^, 60^o^, 50^o^ and 40^o^) (Table 1).

**Table 1:** Values of measurements (A), (B) and (C) by angle in study population in all types of roots (n=60).

Points	Angles	Mean	Standard Deviation	Minimum Value	Maximum Value	P-value**
A	T_1_ (70^o^)	3.98	0.92	2.20	6.30	0.25
	T_2_ (60^o^)	4.08	0.99	2.30	6.60	
	T_3_ (50^o^)	4.22	1.10	2.10	6.60	
	T_4_ (40^o^)	4.36	1.29	2.20	8.90	
B	T_1_ (70^o^)	3.86	0.79	2.30	5.40	0.47
	T_2_ (60^o^)	3.88	0.84	2.20	5.50	
	T_3_ (50^o^)	3.94	0.89	1.80	5.90	
	T_4_ (40^o^)	4.09	1.00	1.70	6.30	
C	T_1_ (70^o^)	4.35	0.90	2.40	6.40	0.39
	T_2_ (60^o^)	4.38	0.91	2.40	6.70	
	T_3_ (50^o^)	4.45	0.97	2.40	6.90	
	T_4_ (40^o^)	4.63	1.15	2.50	7.70	

(A) 2mm, (B) 4mm and (C) 6mm from the tip of the miniscrew. ** ANOVA, p < 0.05.

**Figure 4:**
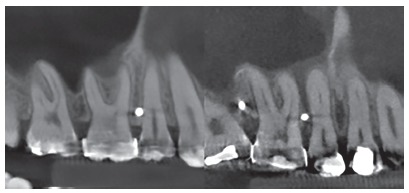
Convergent roots.

**Figure 5:**
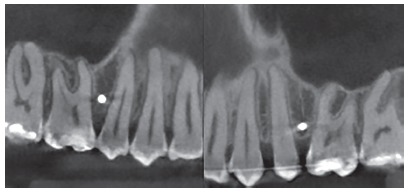
Divergent roots.

**Figure 6:**
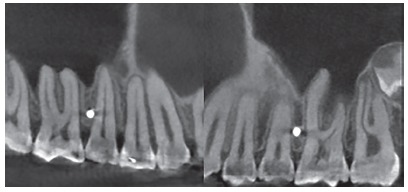
Parallel roots.

During CBCT evaluation the different shapes and dispositions of the roots were observed, which resulted in distinct interradicular spaces. From this observation, three groups were created, which presented interradicular spaces with different characteristics, based on the disposition of the maxillary first molar roots in relation to those of the second premolars. The groups were denominated as follows, regarding roots: convergent, divergent and parallel. The convergent roots, composed of 5 (8.33%) tomographies, presented a reduction in interradicular space in the direction towards the apices, due to convergence of the first molar roots in the direction of the premolar roots (Fig 4). The divergent roots, composed of 21 (35%) tomographies, presented a constant and significant increase in interradicular space in the direction towards the apices (Fig 5). The parallel roots, composed of 34 (56.66%) tomographies, presented an interradicular space that remained equal in the direction towards the apex, however, with a increase in this space only in the apical third (Fig 6). 

Evaluations of the spaces between the roots at the four angulations were made in the four different groups: Group 1, with all the tomographies without distinction of the types of roots; Group 2, with convergent roots; Group 3, with divergent roots; Group 4, with parallel roots.

The CBCT were numbered in alphabetical order according to the patient’s first name. When there were two tomographies for a patient, one of each side, then the first to be numbered was the right side, and then the left side. Thus, all the tomographies had a number. For the intra-examiner test of agreement, the following tomography numbers were used: 10, 20, 30, 40, 50 and 60. All measurements were taken twice by the same operator blinded to group status, with an interval of ten days.^25^ For the agreement test, the Kappa statistics were calculated and obtained 0.91, which indicates excellent agreement between exams. The value varied from 0.89 for the measurement B, 0.91 for the measurement C and 0.94 for the measurement A. 

### Statistical analysis

The variables were descriptively analyzed, and their measurements of central tendency and dispersion were calculated. The Kolmogorov-Smirnov test was used to analyze normality of distribution. After proof of normality of the data, the Student’s-t test was used to identify differences among the groups. In the presence of three or more groups of comparisons, the option was to use the Analysis of Variance (ANOVA), followed by the Tukey test. All the analyses were performed using a level of significance of 95%.

## RESULTS

### Evaluation of Group 1 All the types of roots (n = 60)

The highest obtained values for the distances between roots were for measurement C, followed by A and then B, considering all types of roots and the subdivision by angles (Table 1). This first evaluation only registered the fact that in the middle portion of the miniscrew, which is found at approximately 4mm from the cortical (bone), there is the region with the smallest space between the roots. In the evaluation of Group 1 (all the roots) at different angles, there was a clear trend towards increase in the values from T_1_ to T_4_, although this increase was not statistically significant (Table 2).

**Table 2:** Values of measurements (A), (B) and (C) by angle in a samples with convergent roots (n=05), divergent roots (n=21) and parallel roots (n=34).

Roots	Points	Angles	Mean	Standard Deviation	P-value**
Convergent	A	T_1_ (70^o^)	2.98	0.67	0.99
		T_2_ (60^o^)	2.92	0.69	
		T_3_ (50^o^)	2.88	0.62	
		T_4_ (40^o^)	2.92	0.30	
	B	T_1_ (70^o^)	3.06	0.32	0.70
		T_2_ (60^o^)	2.98	0.28	
		T_3_ (50^o^)	2.76	0.58	
		T_4_ (40^o^)	2.78	0.62	
	C	T_1_ (70^o^)	3.80	0.72	0.99
		T_2_ (60^o^)	3.72	0.66	
		T_3_ (50^o^)	3.72	0.71	
		T_4_ (40^o^)	3.78	0.77	
Divergent	A	T_1_ (70^o^)	4.59	0.89	0.07
		T_2_ (60^o^)	4.80	0.93	
		T_3_ (50^o^)	5.05	0.97	
		T_4_ (40^o^)	5.40	1.29	
	B	T_1_ (70^o^)	4.45	0.66	0.31
		T_2_ (60^o^)	4.52	0.70	
		T_3_ (50^o^)	4.63	0.72	
		T_4_ (40^o^)	4.86	0.87	
	C	T_1_ (70^o^)	4.84	0.87	0.50
		T_2_ (60^o^)	4.85	0.90	
		T_3_ (50^o^)	4.98	0.94	
		T_4_ (40^o^)	5.25	1.20	
Parallel	A	T_1_ (70^o^)	3.75	0.74	0.79
		T_2_ (60^o^)	3.81	0.74	
		T_3_ (50^o^)	3.91	0.86	
		T_4_ (40^o^)	3.92	0.86	
	B	T_1_ (70^o^)	3.62	0.67	0.63
		T_2_ (60^o^)	3.62	0.70	
		T_3_ (50^o^)	3.68	0.69	
		T_4_ (40^o^)	3.81	0.76	
	C	T_1_ (70^o^)	4.13	0.83	0.68
		T_2_ (60^o^)	4.19	0.83	
		T_3_ (50^o^)	4.24	0.89	
		T_4_ (40^o^)	4.39	1.02	

(A) 2mm, (B) 4mm and (C) 6mm from the tip of the miniscrew. ** ANOVA, p < 0.05.

### Evaluation of Group 2 - convergent roots (n = 5)

In the evaluation of Group 2 (convergent roots) at different angles, there was a trend towards reduction in the values from T_1_ to T_4_, although this reduction was not statistically significant. The cases of convergent roots, composed of 5 tomographies, 8.33% of the sample of 60 tomographies, on an average, presented a discrete trend towards reduction in interradicular space, when the angle was diminished; however, it was not statistically significant (Table 2). 

### Evaluation of Group 3 - divergent roots (n = 21)

In the evaluation of Group 3 (divergent roots) at different angles, there was a trend towards increase in the values from T_1_ to T_4_, although this increase was not statistically significant. The cases of divergent roots, composed of 21 tomographies, 35% of the sample of 60 tomographies, on an average, presented a trend towards increase in interradicular space, particularly at Point A, when the angle was diminished. However, it was not statistically significant (Table 2).

### Evaluation of Group 4 - parallel roots (n = 34)

In the evaluation of Group 4 (parallel roots) at different angles, there was a slight trend towards increase in the values from T_1_ to T_4_, although this increase was not statistically significant. The cases of parallel roots, composed of 34 tomographies, 56.66% of the sample of 60 tomographies, on an average, presented a slight trend towards increase in interradicular space when the angle was diminished. However, it was not statistically significant (Table 2).

The results of the evaluation of the four groups were very similar. There were cases in which an increase in space occurred when the insertion angle was reduced. However, there were also simulations in which the space did not change, and in other cases a reduction in space occurred. However, on an average, this trend towards increase or reduction was not statistically significantly. The interradicular spaces along the miniscrew did not increase similarly in all cases in which the angulation was changed from 70^o^ to 40^o^. The distances between the roots varied differently according to the region of the miniscrew body. 

## DISCUSSION

This study evaluated miniscrews angulation in the space between the first molars and second premolars because, according to several authors, this is a region where there is greater availability of space in the maxilla from the vestibular side, which present signifcant clinical application.^9^ ^,^ ^24^

The choice of angulation is not unanimous. Some authors have recommended miniscrew insertion into the maxilla with an angulation of 30^o^ to 40^o^ in relation to the long axis of the tooth, with the purpose of minimizing the risks of contact of the screw with the roots.^9^ ^-^ ^11^ However, other authors have recommended more perpendicular miniscrew insertion into the maxilla, because of the understanding that this factor does not increases the risk of screw contact with the root, and also provides better distribution of force on the cortical bone.^8^ ^,^ ^16^ ^,^ ^17^

The present study recorded the fact that in the middle portion of the miniscrew, which is found at approximately 4mm from the cortical (bone), there is the region with the least space between the roots. Thus, when introducing the miniscrew into the maxilla from the vestibular side, the moment of greatest risk was when the body of the miniscrew was passing through this region, which is equivalent to half of its length when using a screw with a body length of 8mm. One must pay attention to the patient’s sensitivity or to an increase in resistance to the insertion when 4 to 5mm of the miniscrew body are intraosseously inserted. 

When miniscrews were inserted at an angle of 50^o^ and 40^o^, there was superimposition of the virtual screw body on the maxillary sinus in 24 tomographies, equivalent to 40% of the total number of evaluations. This fact must be avoided, because it may lead to complications such as sinusitis and mucosal retention cysts.^18^ ^,^ ^22^ Moreover, screw insertion in a more apical and angled position not only increases the risk of contact with the maxillary sinus, but also increases the risk of sliding during its insertion.^18^

Our results differ from other findings^11^ conducted in typodont teeth. To evaluate the efficiency of more angulated miniscrew insertion in contrast with the more vertical insertion, the methods of the study conducted in typodont teeth should use mannequins with different shapes and dispositions of roots and vestibular cortical (bone). When the evaluation is made in a typodont with divergent roots, it should produce different clinical results from those of simulations performed in mannequins which perhaps present convergent roots. In this study,^11^ only one type of typodont was used, leading to an analysis of the efficiency in only that clinical situation, which may differ from others. Other studies in mannequins must be conducted with a minimum of three clinical situations of roots disposition (convergent roots, divergent roots and parallel roots), in order to be compared with studies carried out with tomographies.

Although the present study suggests that in a region with greater interradicular space, the angulation is not a determinant factor in the positioning of the miniscrew, clinically, this change could modify the height of the line of force action and, consequently, the orthodontic mechanics.

Reduction in the angle of placement during miniscrew insertion, with the purpose of diminishing the risks of contact of the body with the root was not shown to be efficient, considering that in 40% of the virtual miniscrews inserted at an angulation of 40^o^ and 50^o^, the body was superimposed on the maxillary sinus. 

Evaluation of the interradicular spaces at the three points on the miniscrew (A, B and C), performed in the four groups of types of roots (general, convergent, divergent and parallel), at four angulations (70^o^, 60^o^, 50^o^and 40^o^), was rather similar. On an average this trend towards increase or reduction in interradicular space was not statistically significantly. 

## CONCLUSIONS

The change in insertion angle is not a determinant factor in the positioning of miniscrews body in a region with larger interradicular space in posterior maxilla.
